# Topical haemostatic agents for skin wounds: a systematic review

**DOI:** 10.1186/1471-2482-11-15

**Published:** 2011-07-12

**Authors:** Marieke D Groenewold, Astrid J Gribnau, Dirk T Ubbink

**Affiliations:** 1 Department of Quality Assurance & Process Innovation, Academic Medical Center, Amsterdam, The Netherlands; 2 Department of Surgery, Havenziekenhuis, Rotterdam, The Netherlands; 3 Departments of Quality Assurance & Process Innovation and Surgery, Academic Medical Center, Amsterdam, The Netherlands

## Abstract

**Background:**

Various agents and techniques have been introduced to limit intra-operative blood loss from skin lesions. No uniformity regarding the type of haemostasis exists and this is generally based on the surgeon's preference. To study the effectiveness of haemostatic agents, standardized wounds like donor site wounds after split skin grafting (SSG) appear particularly suitable. Thus, we performed a systematic review to assess the effectiveness of haemostatic agents in donor site wounds.

**Methods:**

We searched all randomized clinical trials (RCTs) on haemostasis after SSG in Medline, Embase and the Cochrane Library until January 2011. Two reviewers independently assessed trial relevance and quality and performed data analysis. Primary endpoint was effectiveness regarding haemostasis. Secondary endpoints were wound healing, adverse effects, and costs.

**Results:**

Nine relevant RCTs with a fair methodological quality were found, comparing epinephrine, thrombin, fibrin sealant, alginate dressings, saline, and mineral oil. Epinephrine achieved haemostasis significantly faster than thrombin (difference up to 2.5 minutes), saline or mineral oil (up to 6.5 minutes). Fibrin sealant also resulted in an up to 1 minute quicker haemostasis than thrombin and up to 3 minutes quicker than placebo, but was not directly challenged against epinephrine. Adverse effects appeared negligible. Due to lack of clinical homogeneity, meta-analysis was impossible.

**Conclusion:**

According to best available evidence, epinephrine and fibrin sealant appear superior to achieve haemostasis when substantial topical blood loss is anticipated, particularly in case of (larger) SSGs and burn debridement.

## Background

Limitation of intra-operative blood loss from skin lesions is an important aspect of surgical procedures [1]. Effective and fast haemostasis results in less time spent in the operating room, a more favourable outcome for the patient being under anaesthesia, and an uneventful wound healing process. When substantial blood loss is encountered, haemostasis can play a crucial role in avoiding major haemostatic disturbances [2].

In order to minimize blood loss, for example in patients with large burns after excision of these burns, several treatment options have been introduced such as intravenous vasoconstrictive agents, tourniquets, topical haemostatic agents or subcutaneous agents (i.e. the tumescent technique, frequently used for varicectomy or suction lipectomy) [3-7].

Unfortunately, haemostasis is an understudied subject in the surgical field, and current practice is based on beliefs and habits rather than on evidence [8]. In other words, a so-called 'gold standard' regarding topical haemostasis does not exist. Recently published reviews all conclude that multiple factors determine the agent of choice, including familiarity with the products, patient characteristics and costs [2,8]. Hence, the best choice for achieving haemostasis remains unclear.

For this reason, we decided to investigate the available high-level evidence from the medical literature on all topically applied haemostatic agents. To best appreciate their effects without interference from different types of wound, we chose a quite standardized wound type, i.e. donor sites after split-skin grafting (SSG) as the site of intervention. SSG is a widely used procedure by different surgical specialists. It is frequently used to cover wounds due to various causes, such as (burning) trauma, chronic ulceration, or as part of plastic surgical procedures [3,9]. Although most donor sites are small and do not necessarily require haemostatic treatment, they are well suitable for the examination of superficial bleeding and the response to haemostatic agents applied.

Although several comparative studies on haemostasis of donor sites have been reported, very few showed significant outcomes [4-7]. This was mainly due to small populations, incomparable treatments, and other forms of bias. Hence, the primary aim of this study was to summarize all available strong evidence for the best way(s) for haemostasis in patients with donor sites of SSGs.

## Methods

The conduct and reporting of this systematic review have been performed according to the PRISMA statement [10].

### Search Strategy

We searched MEDLINE, Cochrane Database, and EMBASE up to January 2011 together with our clinical librarian. Keywords used were: (skin transplant* OR (skin transplantation[MeSH] OR (skin graft* OR skin graft[MeSH]))) AND (((hemostasis[MeSH]) OR (hemostatic[MeSH]) OR (haemostatic[MeSH])) OR (hemosta*)). No limits as to language or publication status were applied.

Two reviewers (MG&AG) independently scanned the retrieved abstracts for relevant studies. To be included, articles had to show: (1) the way of achieving haemostasis of the donor site during or after split skin harvest, and (2) a randomised clinical trial (RCT). Exclusion criteria were: clinical comparative studies, case series, case reports, letters, or abstracts. In case of disagreement between the two reviewers, a third reviewer (DU) was involved.

### Haemostatic Agents, Outcome Measures, and Data Analysis

All agents or techniques used to reduce blood loss from donor sites were included.

Results of each trial were examined with regard to four outcome measures: blood loss (reported using different parameters, e.g. amount of blood (products) in gauzes, swabs or filters, time to haemostasis, use of electrocautery, need for blood transfusions), donor site wound healing, adverse effects, and costs.

Data were entered into Revman (version 5.0.23, Cochrane Collaboration) and 95% confidence intervals (CI) were calculated for every comparison. For continuous outcomes, mean differences (MD) were calculated. For dichotomous outcomes, risk ratios (RR) and numbers needed to treat (NNT) were used.

### Assessment of Methodological Quality

Methodological quality of the RCTs was assessed using 'The Cochrane Collaboration's Tool for Assessing Risk of Bias' [11]. Again, this assessment was made by two reviewers independently (MG&AG).

## Results

### Description of trials

**Figure 1:**
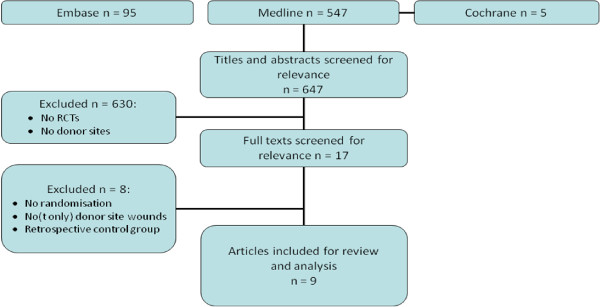
**Literature search and article selection**. RCTs; randomised clinical trials.

We found 647 potentially relevant titles. After screening of titles or abstracts 17 articles matched our inclusion criteria. Full texts were screened and eventually, 9 relevant articles were included. Study inclusion and reasons for exclusion are summarised in Figure 1. Trial sizes ranged from 5 to 56 patients totalling 376 donor sites in 340 (mainly burn) patients. In five trials donor sites were divided in two equivalently sized halves, while patients functioned as their own controls [12-16]. Three trials [17-19] compared entire donor sites, while one trial [20] only included patients with two similar donor sites (i.e. opposite thighs). In one trial, the haemostatic effect of alginate dressings was investigated. This possible effect has been ascribed to their calcium content, being an important element in the coagulation cascade [21]. Trial characteristics are summarised in table 1. Due to a large variability of outcome measurements and comparisons, no meta-analyses could be performed.

**Table 1:** Characteristics of included studies

Trial	Patients	Intervention	Comparison	Outcomes
Barret JP, 1999	42 pediatric burn patients (21 vs. 21)	Epinephrine 1:10.000 (and thrombin 1:300.000) (topical)	Saline (and thrombin)	Blood loss in ml and ml/cm^2^, Ht, Hb up to 8 hours post-operatively, blood transfusions

Brezel BS, 1987	32 burn patients (32 vs. 32)	Epinephrine 1:200.000 (topical)	Thrombin	Blood loss visually estimated from photographs 5 minutes post-operatively

Gacto P, 2008	56 burn patients (25 vs. 31)	Epinephrine-lidocaine Subcutaneous 1:500.000	Saline	Overall blood loss visually estimated, use of electrocautery, days hydrocolloid maintained

Netscher DT, 1996	52 patients (12 vs. 8 vs. 12 vs. 7 vs. 13)	Epinephrine/K-Y jelly 1:50.000 (topical)	Epinephrine spray, thrombin, K-Y jelly, mineral oil.	Time to haemostasis (minutes)

Carucci DJ, 1984	24 patients (6 vs. 6 vs. 12)	Phenylephrine 1:20.000 (topical)	Thrombin	Blood loss measured by Hb in paper disks

Greenhalgh DG, 1999	34 burn patients (<15% TBSA) (34 vs. 34)	Fibrin sealant (duoflo Y-shaped adapter)	Placebo	Blood loss visually estimated and measured by Hb in sponges, donor site healing viewed on photographs

Nervi C, 2001	61 burn patients (donor site 2-8% TBSA) (61 vs. 61)	Fibrin sealant (duoflo Y-shaped adapter)	Placebo	Time to haemostasis

Drake DB, 2003	34 patients with SSG areas 5-15 cm (34 vs. 34)	Fibrin sealant (Vivostat)	Thrombin	Time to haemostasis (seconds)

Steenfos HH, 1998	5 patients (7 donor sites) (7 vs. 7)	Alginate dressing	Fine mesh gauze	Blood loss measured by iron content of dressings

### Risk of bias in included trials

**Figure 2:**
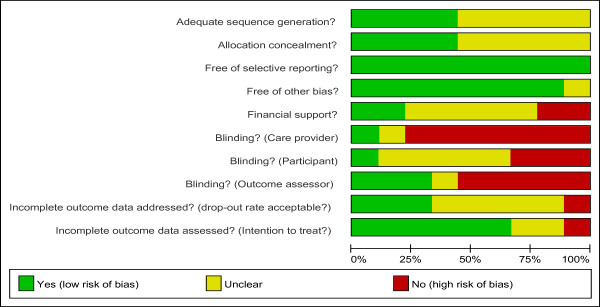
**Risk of bias table showing the methodological quality assessment of the 9 included RCTs**.

Methodological quality was generally fair and is summarised in figure 2. Study groups also appeared comparable at baseline. None of the included trials used a power analysis to calculate the minimal sample size required to achieve statistical significance for the estimated treatment effect.

### Effects of interventions (see table 2)

**Table 2:** Effects of interventions sorted by comparison.

Comparison	Trial	Haemostatic effect	Healing	Adverse effects	Costs
1. **Epinephrine vs. thrombin**	Brezel et al. 1987	+	NS	NS	direct costs in favour of epinephrine
	
	Carucci et al. 1984	+	no data	NS	probably favouring epinephrine
	
	Netscher et al. 1996	+	no data	NS	direct costs in favour of epinephrine*

2. **Epinephrine vs. control**	Carucci et al. 1984	+	no data	NS	probably favouring control*
	
	Gacto et al. 2008	+	favouring epinephrine	NS	no data
	
	Netscher et al. 1996	+	no data	NS	probably favouring control*
	
	Barret et al. 1999	NS	NS	NS	no data

3. **Fibrin sealant vs. control**	Nervi et al. 2001	+	no data	no data	no data
	
	Greenhalgh et al. 1999	+/NS/NS**	NS	NS	no data

4. **Fibrin sealant vs. thrombin**	Drake et al. 2003	+	NS	NS	no data

5. **Thrombin vs**. **control**	Carucci et al. 1984	+	no data	NS	probably favouring control*
	
	Netscher et al. 1996	+	no data	NS	probably favouring control*

6. **Alginate dressing vs. fine mesh gauze**	Steenfos et al. 1998	+	NS	NS	no data

+ = significantly in favour of the former agent;

- = significantly in favour of the latter agent;

NS = not significant.

* Only direct costs per product or per patient were given, so no conclusion on overall costs per treatment could be conducted.

** Blood loss was estimated in three different ways.

## Comparison 1. Epinephrine vs. thrombin (3 trials)

### 1.1 Haemostatic Effect

#### 1.1.1 Blood loss

Brezel et al. reported less bleeding was visually stated at epinephrine treated sites in significantly more patients (29 out of 32), than at thrombin treated sides (0 out of 32) (RR 59.00; 95%CI 3.76 to 925.91; NNT = 2). Size of grafted areas was not reported[13].

Carucci et al. reported that the amount of blood absorbed was significantly less from donor sites treated with phenylephrine than with thrombin (p < 0.05) [14]. We had no access to original data and therefore could not check this result. All assessed donor sites were 200 cm^2^.

#### 1.1.2 Time to haemostasis

Netscher et al. compared two epinephrine-containing agents to thrombin [19]. Both epinephrine/K-Y jelly (MD -2.30; 95%CI -3.83 to -0.77 minutes) and epinephrine spray (MD -2.19; 95%CI -4.03 to -0.35 minutes) resulted in significantly quicker haemostasis than treatment with thrombin. Size of grafted areas ranged from 15 to 891 cm^2^.

### 1.2 Adverse effects

All three trials described that adverse or systemic effects due to treatment with epinephrine or thrombin did not occur.

### 1.3 Costs

Brezel et al. reported that use of epinephrine compared to thrombin would save the hospital US$9.85, and the patient US$19.50 per grafting procedure[13].

Carucci et al. reported costs for haemostatic treatment per patient was US$3 for treatment with phenylephrine, and US$92 for treatment with thrombin [14]. Both studies reported on direct costs without statistical analysis.

Netscher et al. only reported general product costs, and stated that thrombin spray was by far most expensive [19].

## Comparison 2. Epinephrine vs. control (saline/mineral oil/K-Y jelly; 4 trials)

### 2.1 Haemostatic Effect

#### 2.1.1 Blood loss

Gacto et al. found a significant difference in blood loss, when comparing visually estimated blood loss from donor site areas [18]. Blood loss was estimated as "less than normal" in 29 (out of 31) cases treated with epinephrine, compared to 9 (out of 25) cases treated with saline (RR: 2.60; 95%CI 1.53 to 4.42; NNT = 2). Also electrocautery was significantly less used after application of epinephrine; 2 versus 16 patients (RR 0.10; 95%CI 0.03 to 0.40). Additionally, hydrocolloid dressings remained in situ significantly more days after treatment with epinephrine (MD 3.55; 95%CI 2.63 to 4.47). The mean donor site size was 3.9% (SD 2%) of total body surface (TBSA).

Carucci et al. reported that amount of blood absorbed in donor sites treated with phenylephrine was significantly less than in those treated with saline (p < 0.05) [14]. The size of all assessed donor sites was 200 cm^2^.

Barret et al. reported no significant difference in blood loss (MD -228.00; 95%CI -908.51 to 452.51 ml). Even when corrected for grafted area (ml/cm^2^) the difference remained insignificant (MD -0.02; 95%CI -0.11 to 0.05) [15]. No significance in the number of blood transfusions (RR 0.75; 95%CI 0.19 to 2.95) was found. Sizes of grafted areas ranged from 0 to 5000 cm^2^.

#### 2.1.2 Time to haemostasis

Netscher et al. compared epinephrine spray and epinephrine/K-Y jelly mixture to mineral oil and K-Y jelly alone [19]. With both epinephrine-containing agents, haemostasis was achieved significantly faster than with control agents. Epinephrine spray was faster than mineral oil (MD -6.25; 95%CI -7.88 to -4.62 minutes) and K-Y jelly (MD -5.00; 95%CI -7.38 to -2.62 minutes). Epinephrine/K-Y jelly mixture was also faster than mineral oil (MD -6.36; 95%CI -7.63 to -5.09 minutes) and K-Y jelly alone (MD -5.11; 95%CI -7.27 to -2.95 minutes). The size of grafted area ranged from 15 to 891 cm^2^.

### 2.2 Healing

Gacto et al. reported the proportion of re-epithelialised skin after 1 week was 98.5% in the group treated with epinephrine, and 71% in the group treated with saline [18].

Netscher et al. did not report any (negative) effect on wound healing [19]. Barret et al. reported comparable wound healing of donor sites in both study groups [17].

### 2.3 Adverse effects

No differences in complications, side effects, or adverse events were reported in any trial.

## Comparison 3. Fibrin sealant vs. control (2 trials)

### 3.1 Haemostatic Effect

#### 3.1.1 Blood loss

Greenhalgh et al. reported that estimated blood loss by registering blood-soaked swabs did not result in a significant difference (MD -5.00; 95%CI -128.37 to 118.37 ml) between the fibrin sealant group 245 ± 283 ml) and the control group (250 ± 280 ml) [20]. Additionally, no significant differences were found in haemoglobin concentrations measured in laparotomy sponges (n = 41) that were placed on donor sites during 1 minute postoperatively (MD -0.03; 95%CI -0.29 to 0.23 absorbance value), and during following 5 minutes postoperatively (MD -0.12; 95%CI -0.47 to 0.23 absorbance value). Absorbance values were determined at 540 nm from a solution of eluted haemoglobin into 1 litre 0.007 M ammonium-hydroxide. Mean size of grafted area was 5.5% TBSA (ranging from 1 to 14.8%).

#### 3.1.2 Time to haemostasis

Nervi et al. reported less mean time (seconds) to haemostasis after treatment with fibrin sealant (193 ± 131 s), compared to no treatment (392 ± 153 s) (MD -199; 95%CI -249.55 to -148.45 s) [15]. Mean size of grafted areas was 3.4% TBSA (SD 1.2%).

### 3.2 Healing

Greenhalgh et al. reported no differences in rates of wound healing between both groups [20]. They also compared eventual cosmetic appearance. No significant differences were noted; 94.1% of the fibrin sealant group was ranked as good/excellent compared to 89.3% of the control group.

### 3.3 Adverse effects

Greenhalgh et al. reported one adverse event that was due to use of fibrin sealant (graft loss by excessive application of fibrin sealant) [20]. However, this referred to an adverse effect located on the recipient site. When fibrin sealant is exclusively used for haemostasis, this adverse effect does not appear of great importance.

## Comparison 4. Fibrin sealant vs. thrombin (1 trial)

### 4.1 Haemostatic Effect

#### 4.1. Time to haemostasis

Drake et al. recorded significantly less time (seconds) to haemostasis in areas treated with fibrin sealant (mean 68, range 5 to 398 s), than with thrombin (mean 135, range 22 to 601 s) (MD -67.00; 95%CI -126.84 to -7.16 s). Sizes of grafted areas were not reported [12].

### 4.2 Adverse effects

No adverse effects were considered related to fibrin sealant use [12].

## Comparison 5. Thrombin vs. control (saline/mineral oil/K-Y jelly; 2 trials)

### 5.1 Haemostatic Effect

#### 5.1.1 Blood loss

Carucci et al. reported blood absorbed from donor sites treated with thrombin was significantly less than from sites treated with saline (p < 0.05) [14]. The size of all grafted areas was 200 cm^2^.

#### 5.1.2 Time to haemostasis

Netscher et al. reported that haemostasis is achieved significantly faster (in minutes) after treatment with thrombin, than with mineral oil (MD -4.06; 95%CI -5.81 to -2.31), or K-Y jelly (MD -2.81; 95%CI -5.28 to -0.34). Size of grafted area ranged from 15 to 891 cm^2 ^[19].

### 5.2 Healing

Netscher et al. reported uniform wound healing among study groups measured by observation, but no data were reported [19].

### 5.4 Costs

Carucci et al. reported costs for haemostatic treatment with thrombin were US$92 per patient, but those for saline were not mentioned [14].

Netscher et al. only stated that thrombin spray was by far the most expensive product [19]. They did not report costs per patient or per treatment.

## Comparison 6. Alginate dressing vs. fine mesh gauze (1 trial)

### 6.1 Haemostatic Effect

#### 6.1.1 Blood loss

Steenfos et al. reported a significantly higher amount of blood absorption 10 minutes postoperatively by alginate dressings (μg Fe/cm^2 ^wound area) (mean 1110, SD 262), compared to mesh gauze dressings (mean 774, SD 314) (MD -336; 95%CI -638.95, -33.05) [16]. The average size of donor sites was 105 cm^2^.

### 6.2 Healing

No significant differences were reported in number of donor sites completely epithelialised at day 6 between sites treated with alginate (9 out of 22) and mesh gauze (7 out of 22) (RR 1.29; 95%CI 0.58 to 2.84) [16].

### 6.3 Adverse effects

They also reported no complications with either treatment [16].

## Discussion

This systematic review shows evidence for the haemostatic effect of epinephrine, fibrin sealant, thrombin, and alginates on donor sites after split-skin grafting. In particular, epinephrine and fibrin sealant seem to be superior over other haemostatic products or placebo to reduce blood loss. These agents can shorten time to haemostasis with about 3 to 6 minutes. However, costs of the use of these agents are poorly investigated, as well as their effect on wound healing. This weakens the possibility to decide for a certain haemostatic agent based on the evidence presently available.

Unfortunately, no trial directly compared epinephrine to fibrin sealant. Hence, it remains unclear which of the two agents is preferable for haemostasis. Epinephrine and fibrin sealant appear superior to thrombin, which per se has better haemostatic properties than mineral oil, K-Y jelly, or saline. Effect sizes were mainly described in terms of minutes to haemostasis rather than an accurate appreciation of the amount of blood loss. This time gain seems subordinate to the actual blood loss. Anyhow, use of these agents appears helpful when large skin transplants are needed or considerable blood loss is anticipated, for example in burn wound resections.

Only four out of nine trials presented results on cosmetic appearance or wound healing, which may have been hampered by prolonged blood or fluid loss from the donor site. No trial showed any adverse effects of local haemostatics, in particular no systemic adrenergic side effects due to epinephrine, as described previously by Hughes et al. [8]. Considering costs of treatment, epinephrine seems less expensive than thrombin and fibrin sealant, but evidence and uniformity on this matter is lacking.

Recently, newly developed haemostatic agents, such as platelet gel, CoSeal^®^, BioGlue^® ^or Ostene^® ^were introduced [22,23], but were not included in our systematic review, because no randomized controlled trials were performed yet comparing these agents in skin lesions.

The evidence obtained has some limitations. First of all, some trials used rather vague and subjective outcome measurements, while explicit recording and reporting of adverse effects was rare. Two trials assessed blood loss by visually estimating the amount of blood, which is imprecise and sensitive to bias. Although results were significant, clinical relevance remains questionable. Second, blinding of care providers (surgeons and nurses, who usually also assess the outcome) was difficult to accomplish because of the differing application of haemostatic agents (different gauzes, injections, applicators). In most trials patients were probably blinded, because they are unconscious during the SSG procedure, and are likely not to be informed about which haemostatic treatment they received. However, three trials specifically reported blinding of the outcome assessor(s). This is the best option to reduce the risk of bias if blinding of caregivers and patients is impossible.

In a comprehensive (but not systematic) review, Achneck et al. [22] discussed the mechanism of action, (dis)advantages, and recommendations for use of multiple (new) topical haemostatic agents. Unfortunately, part of their evidence was based on case series and case reports, having a high risk of bias. The authors recommended several agents for different procedures. They conclude that the ideal haemostatic agent does not exist and the agent of choice depends on several aspects such as type of procedure, mechanism of action, and patient characteristics. According to the present review of efficacy, adverse effects, and healing properties, epinephrine and fibrin sealant come close to being ideal. Furthermore, epinephrine is relatively inexpensive. In another review, Samudrala et al. [23] describe principles of haemostasis and mechanisms of action of several agents. They conclude, based also on non-comparative studies, that use of haemostatic agents in general contributes to faster patient recovery time, avoids adverse events, and reduces overall procedure time. This supports the evidence found in our review, although effect sizes differ greatly among different agents. In addition, familiarity with these products and their preparation is a prerequisite for optimal use and to improve patient outcomes.

In order to determine the single most effective agent for haemostasis, further investigation is required. For this purpose, blood loss should be reported in an objective and precise manner. Moshaver et al. [24] measured blood loss during endoscopic sinus surgery using a 'standardized scale': evaluating blood loss by the need of electrocautery. Even though such definitions are detailed, its applicability still remains questionable. The best method of assessing wound healing is also unclear. The most accurate and objective method used by Steenfos et al. [16] consisted of assessing punch biopsies by a pathologist masked for the treatment given. Although this method leaves little subjectivity, it informed only on healing speeds, not on quality.

## Conclusions

Haemostatic agents are particularly useful for patients requiring larger split skin graft harvests, burn wound debridement, or for other reasons why minimisation of (topical) blood loss is desired. According to best available evidence as summarised in this review, epinephrine and fibrin sealant appear superior agents for achieving quick and effective haemostasis. Both agents reduce the amount and the time of bleeding, while they do not seem to impair wound healing or lead to other adverse events. It remains unclear which of these two agents is to be preferred as to healing quality, safety, and costs. A well-performed randomised clinical trial comparing at least epinephrine and fibrin sealant is desirable to produce high-quality and clinically relevant results. For this purpose we recommend the use of clear outcome measures (including side effects, wound healing, and safety), blinded outcome assessors, and an economic evaluation.

## Competing interests

None of the authors have any (non-)financial interest linked to the outcomes of this study. This study was funded by an unrestricted grant from the Dutch Burns Foundation [25].

## Authors' contributions

MG and AJG performed the search, data collection and analysis, and wrote the manuscript. DU conceived the study and critically reviewed, and revised the manuscript. All authors have read and approved the final manuscript.

## Pre-publication history

The pre-publication history for this paper can be accessed here:

http://www.biomedcentral.com/1471-2482/11/15/prepub

## References

[B1] Palm MD Altman JS Topical Hemostatic Agents: A Review Dermatol Surg 2008 34 431 445 10.1111/j.1524-4725.2007.34090.x 18248471

[B2] Niemi T Svartling N Syrjala M Asko-Seljavaara S Rosenberg P Haemostatic Disturbances in Burned Patients During Early Excision and Skin Grafting Blood Coagul Fibrinolysis 1997 9 19 28 10.1097/00001721-199801000-00003 9607115

[B3] Andreassi A Classification and pathophysiology of skin grafts Clin Dermatol 2005 23 332 337 10.1016/j.clindermatol.2004.07.024 16023927

[B4] Achauer BM Miller SR Lee TE The Hemostatic Effect of Fibrin Glue on Graft Donor Sites J Burn Care Rehabil 1994 1 24 28 10.1097/00004630-199401000-00005 8150838

[B5] Robertson RD Bond P Wallace B Shewmake K Cone J The Tumescent technique to significantly reduce blood loss during burn surgery Burns 2001 27 835 838 10.1016/S0305-4179(01)00057-2 11718986

[B6] Hughes WB DeClement FA Hensell DO Intradermal injection of epinephrine to decrease blood loss during split-thickness skin gafting J Burn Care Rehabil 1996 17 243 245 10.1097/00004630-199605000-00011 8736371

[B7] Mzezewa S Jonsson K Aberg M Sjoberg T Salemark L A Prospective double blind randomized study comparing the need for blood transfusion with terlipressin or a placebo during early excision and grafting of burns Burns 2004 30 236 240 10.1016/j.burns.2003.11.004 15082350

[B8] Barnard J Millner R A Review of Topical Hemostatic Agents for Use in Cardiac Surgery Ann Thorac Surg 2009 88 1377 1383 10.1016/j.athoracsur.2009.02.092 19766855

[B9] Geary PM Tierman E Management of split skin graft donor sites - results of a national survey J Plast Reconstr Aesthet Surg 2009 12 1677 1683 10.1016/j.bjps.2008.07.036 19131290

[B10] Moher D Liberati A Tetzlaff J Altman DG The PRISMA Group Preferred Reporting Items for Systematic Reviews and Meta-Analyses: The PRISMA Statement PLoS Med 2009 6 6 e1000097 10.1371/journal.pmed.1000097 PMC2707599 19621072

[B11] Higgins JPT Green S Cochrane Handbook for Systematic Reviews of Interventions 2008 West Sussex: John Wiley & Sons Ltd

[B12] Drake DB Wong LG Hemostatic effect of Vivostat Patient-derived Fibrin Sealant on Split-Thickness Skin Graft Donor Sites Ann Plast Surg 2003 4 367 372 10.1097/01.SAP.0000041484.22953.6D 12671377

[B13] Brezel BS McGeever KE Stein JM Epinephrine v Thrombin for Split-Thickness Donor Site Hemostasis J Burn Care Rehabil 1987 2 132 134 10.1097/00004630-198703000-00009 3294843

[B14] Carucci DJ Pearce RSC Innes DJ Rodehaever GT Kennedy GJ Edlich RF Evaluation of Hemostatic Agents for Skin Graft Donor Sites J Burn Care Rehabil 1984 5 321 323 10.1097/00004630-198407000-00015

[B15] Nervi C Gamelli RL Greenhalgh DG Luterman A Hansbrough JF Achauer BM A Multicenter Clinical Trial to Evaluate the Topical Hemostatic Efficacy of Fibrin Sealant in Burn Patients J Burn Care Rehabil 2001 22 99 103 10.1097/00004630-200103000-00003 11302613

[B16] Steenfos HH Agren MS A fibre-free alginate dressing in the treatment of split thickness skin graft donor sites J Eur Acad Dermatol Venereol 1998 11 252 256 10.1111/j.1468-3083.1998.tb00978.x 9883438

[B17] Barret JP Dziewulski P Wolf SE Desai MH Nichols RJ II Herndon DN Effect of topical and subcutaneous epinephrine in combination with topical thrombin in blood loss during immediate near-total burn wound excision in pediatric burned patients Burns 1999 25 509 513 10.1016/S0305-4179(99)00038-8 10498359

[B18] Gacto P Miralles F Pereyra JJ Perez A Martinez E Haemostatic effects of adrenaline-lidocaine subcutaneous iniltration at donor sites Burns 2008 35 343 347 18950945 10.1016/j.burns.2008.06.019

[B19] Netscher DT Carlyle T Thronby J Bowen D Harris S Clamon J Hemostasis at Skin Graft Donor Sites: Evaluation of Topical Agents Ann Plast Surg 1996 36 7 10 10.1097/00000637-199601000-00002 8722976

[B20] Greenhalgh DG Gamelli R Lee M Delavari M Lynch JB Hansbrough JF Multicenter Trial to Evaluate the Safety and Potential Efficacy of Pooled Human Fibrin Sealant for the Treatment of Burn Wounds J Trauma 1999 46 433 440 10.1097/00005373-199903000-00014 10088846

[B21] Lansdown AB Calcium: a potential central regulator in wound healing in the skin Wound Repair Regen 2002 10 5 271 85 10.1046/j.1524-475X.2002.10502.x 12406163

[B22] Achneck HE Sileshi B Jamiolkowski RM Albala D Shapiro ML Lawson JH A Comprehensive Review of Topical Hemostatic Agents Ann Surg 2010 251 217 228 10.1097/SLA.0b013e3181c3bcca 20010084

[B23] Samudrala A Topical Hemostatic Agents in Surgery: A Surgeon's Perspective AORN J 2008 88 1 11 10.1016/j.aorn.2008.08.012 18790097

[B24] Moshaver A Lin D Pinto R Witterick I The Hemostatic and Hemodynamic Effects of Epinephrine during Endoscopic Sinus Surgery Arch Otolaryngol Head Neck Surg 2009 10 1005 1009 10.1001/archoto.2009.144 19841339

[B25] The Dutch Burns Foundation http://www.brandwonden.nl

